# Clinical outcome of nosocomial pneumonia caused by Carbapenem-resistant gram-negative bacteria in critically ill patients: a multicenter retrospective observational study

**DOI:** 10.1038/s41598-022-11061-7

**Published:** 2022-05-07

**Authors:** Chih-Yu Chen, Kuang-Yao Yang, Chung-Kan Peng, Chau-Chyun Sheu, Ming-Cheng Chan, Jia-Yih Feng, Sheng-Huei Wang, Chia-Min Chen, Zhe-Rong Zheng, Shinn-Jye Liang, Yu-Chao Lin, T-CARE (Taiwan Critical Care and Infection) Group, Chih-Yu Chen, Bing-Ru Wu, Yi-Cheng Shen, Wei-Cheng Chen, Shinn-Jye Liang, Yu-Chao Lin, Kuang-Yao Yang, Jia-Yih Feng, Chung-Kan Peng, Sheng-Huei Wang, Chau-Chyun Sheu, Chia-Min Chen, Ming-Cheng Chan, Zhe-Rong Zheng

**Affiliations:** 1 Division of Pulmonary and Critical Care Medicine, Department of Internal Medicine, China Medical University Hospital, No. 2, Yude Road, Taichung, 404332 Taiwan; 2 Graduate Institute of Biomedical Electronics and Bioinformatics, National Taiwan University, Taipei, Taiwan; 3 Department of Chest Medicine, Taipei Veterans General Hospital, Taipei, Taiwan; 4 Institute of Emergency and Critical Care Medicine, School of Medicine, National Yang Ming Chiao Tung University, Taipei, Taiwan; 5 Cancer Progression Research Center, National Yang Ming Chiao Tung University, Taipei, Taiwan; 6 Division of Pulmonary and Critical Care Medicine, Department of Internal Medicine, Tri-Service General Hospital, National Defense Medical Center, Taipei, Taiwan; 7 Division of Pulmonary and Critical Care Medicine, Department of Internal Medicine, Kaohsiung Medical University Hospital, Kaohsiung Medical University, Kaohsiung, Taiwan; 8 Department of Internal Medicine, School of Medicine,College of Medicine, Kaohsiung Medical University, Kaohsiung, Taiwan; 9 Division of Critical Care and Respiratory Therapy, Department of Internal Medicine, Taichung Veterans General Hospital, Taichung, Taiwan; 10 National Chung Hsing University, Taichung, Taiwan; 11 School of Medicine, National Yang Ming Chiao Tung University, Taipei, Taiwan; 12 Division of Pulmonary Medicine, Department of Internal Medicine, Chung Shan Medical University Hospital, Taichung, Taiwan; 13 School of Medicine, China Medical University, Taichung, Taiwan

**Keywords:** Bacterial infection, Antimicrobial therapy

## Abstract

Nosocomial pneumonia caused by carbapenem-resistant gram-negative bacteria (CRGNB) is a growing threat due to the limited therapeutic choices and high mortality rate. The aim of this study was to evaluate the prognostic factors for mortality in patients with nosocomial pneumonia caused by CRGNB and the impact of colistin-based therapy on the outcomes of intensive care unit (ICU) patients. We conducted a retrospective study of the ICUs in five tertiary teaching hospitals in Taiwan. Patients with nosocomial pneumonia caused by CRGNB from January 2016 to December 2016 were included. Prognostic factors for mortality were analyzed using multivariate logistic regression. The influence of colistin-based therapy on mortality and clinical and microbiological outcomes were evaluated in subgroups using different severity stratification criteria. A total of 690 patients were enrolled in the study, with an in-hospital mortality of 46.1%. The most common CRGNB pathogens were *Acinetobacter baumannii* (78.7%) and *Pseudomonas aeruginosa* (13.0%). Significant predictors (odds ratio and 95% confidence interval) of mortality from multivariate analysis were a length of hospital stay (LOS) prior to pneumonia of longer than 9 days (2.18, 1.53–3.10), a sequential organ failure assessment (SOFA) score of more than 7 (2.36, 1.65–3.37), supportive care with vasopressor therapy (3.21, 2.26–4.56), and escalation of antimicrobial therapy (0.71, 0.50–0.99). There were no significant differences between the colistin-based therapy in the deceased and survival groups (42.1% vs. 42.7%, p = 0.873). In the subgroup analysis, patients with multiple organ involvement (> 2 organs) or higher SOFA score (> 7) receiving colistin-based therapy had better survival outcomes. Prolonged LOS prior to pneumonia onset, high SOFA score, vasopressor requirement, and timely escalation of antimicrobial therapy were predictors for mortality in critically ill patients with nosocomial CRGNB pneumonia. Colistin-based therapy was associated with better survival outcomes in subgroups of patients with a SOFA score of more than 7 and multiple organ involvement.

## Introduction

Nosocomial pneumonia is one of the most common hospital-acquired infections and is associated with significant morbidity and mortality in critically ill patients^1^. Carbapenem-resistant gram-negative bacteria (CRGNB), including *Acinetobacter baumannii* (CRAB), *Pseudomonas aeruginosa* (CRPA), and *Enterobacteriaceae* (CRE), have become an increasingly significant cause of nosocomial pneumonia in many countries^2,3^. When comparing with carbapenem-susceptible gram-negative bacteria (CSGNB), infections caused by CRGNB are associated with more than 2 times the risk of death, both globally and regionally in Taiwan^4–6^. In previous studies, prognostic factors for mortality in patients with nosocomial pneumonia such as presence of septic shock, high sequential organ failure assessment (SOFA) score, and inappropriate empiric antimicrobial therapy have been reported^7,8^; however, there is still minimal data to discuss nosocomial CRGNB pneumonia in intensive care units (ICU).

Due to the minimal effectiveness of antimicrobial agents, nosocomial pneumonia caused by CRGNB presents a major challenge to delivering adequate therapy and is associated with high mortality, ranging from 41 to 53%^9,10^. Despite the recent development of novel antimicrobial agents^11^, polymyxins, most commonly colistin (polymyxin E), remain the most important and frequently used agents against CRGNB^12^. Several issues have been raised around colistin-based therapy to enhance its activity and efficacy, including loading dose^13,14^, maintenance dose^15^, combination therapy^16,17^, and adjunctive inhalation therapy^18,19^. While colistin-based therapy is considered the backbone of the antimicrobial therapy against CRGNB, there is still controversy regarding its mortality benefit over other antimicrobial agents^10,20,21^.

The association of colistin-based therapy with improved survival and its influence on different severity stratification criteria remains to be clarified. The aim of this study was to evaluate the prognostic factors associated with in-hospital mortality in critically ill patients with nosocomial CRGNB pneumonia and the impact of colistin-based therapy on patient outcomes.

## Methods

### Study design

We conducted a retrospective multicenter study of the ICUs in five tertiary teaching hospitals in Taiwan. All consecutive patients who were admitted to the ICU with microbiologically documented nosocomial pneumonia caused by CRGNB between January 2016 and December 2016. Chart review was performed. Patients were excluded if (1) they were younger than 20 years of age, (2) they had lung cancer with post-obstructive pneumonia, (3) they had human immunodeficiency virus infection with a CD4 cell count of less than 200 cells/mm^3^, (4) they did not receive any antimicrobial therapy with curative intent.

### Data collection technique

The data were collected from the medical records of patients, which contained demographic data (age, gender, smoking status, alcohol consumption, source of admission, and type of ICU and supportive care), comorbidities (malignancy, liver disease, including chronic hepatitis or cirrhosis, heart failure, hypertension, cerebral vascular disease, neurodegenerative disorder, chronic kidney disease, chronic lung disease, diabetes mellitus, immunocompromised due to long-term use of steroid or other immunosuppressants, autoimmune disease), clinical manifestations, laboratory findings, severity of illness on the day of pneumonia diagnosis evaluated by the Acute Physiology and Chronic Health Evaluation (APACHE) II score and SOFA score, microbiological profile, antimicrobial therapy, and outcome evaluation.

Semi-quantitative and quantitative cultures were obtained either from bronchoalveolar lavage, endotracheal aspirate, or qualified sputum specimens. Antimicrobial susceptibility tests were performed using either the VITEK® 2 system (bioMérieux, Marcy-l'Etoile, France) or the BD Phoenix™ system (Becton Dickinson Diagnostic Systems, Sparks, MD, USA). Minimum inhibitory concentration (MIC) breakpoints were established based on the Clinical Laboratory and Standards Institute guidelines^22^. All patients received antimicrobial therapy with curative intent. Information about antimicrobial therapy was recorded only if the prescription duration was longer than or equal to 3 days.

### Definitions

Carbapenem-Resistant Gram-Negative Bacteria was defined as *A. baumannii*, *P. aeruginosa,* or *Enterobacteriaceae* with resistance to any carbapenem (intermediately susceptible results were reported as resistant). Pneumonia was considered nosocomial when onset occurred > 48 h after hospitalization. The diagnosis required radiographic images of a new and persistent pulmonary infiltrate and at least one of the following criteria: body temperature > 38 °C or < 36 °C; leukocytosis > 12,000 cells/mm^3^, leukopenia < 4000 cells/mm^3^ or band form > 10%; cough or purulent sputum. Nosocomial pneumonia was considered to be ventilator-associated when it occurred > 48 h after the initiation of mechanical ventilation.

Escalation of therapy was defined as a switch to or addition of any antimicrobial agent with additional coverage after definitive identification of CRGNB. Definitive therapy was defined as antimicrobial therapy that was continued or initiated after antimicrobial susceptibilities were available. Due to the possibility of in vivo activity of carbapenem despite in vitro resistance, we did not adopt the term “inappropriateness of empirical therapy,” which has been well defined and discussed in other publications.

Clinical outcome was classified into three categories: cure (complete resolution of symptoms and signs with no further requirement for antimicrobial therapy), improvement (partial resolution of symptoms and signs with requirement for antimicrobial therapy), and failure (persistence of symptoms and signs or death). Cure and improvement were considered as effective therapeutic outcomes. Microbiological outcome was classified into four categories: eradication (absence of the baseline pathogen in at least two sets of respiratory specimens), persistence (persistence of the baseline pathogen in respiratory specimens), recurrence (re-detection of the baseline pathogen in respiratory specimens within 14 days of eradication), and undetermined (culture of respiratory specimens not available during follow-up).

### Outcome evaluation

We used in-hospital mortality of any cause as the main outcome measure in the study. Other outcome measures included 90-day mortality, clinical cure/improvement, and microbiological eradication at day 14 following pneumonia diagnosis.

### Statistical analysis

Normally- and non-normally distributed continuous data were expressed as mean with standard deviation (SD) and median with interquartile range (IQR), respectively. Categorical variables were reported as number (%). Student’s t-test was used for analysis of continuous variables, and the Chi-square (χ2) test was used for categorical variables. Multivariate analysis using multiple logistic regression was performed to evaluate the prognostic factors associated with in-hospital mortality. Multicollinearity was measured by variance inflation factors, and the fitness of the model was assessed using Hosmer and Lemeshow test. To explore the pattern of organ involvement, k-means clustering was used to derive subtypes based upon individual SOFA subscore data. Internal validation was performed by assessing the silhouette width and Davies-Bouldin score.

We used logistic regression to evaluate the influence of colistin-based therapy on in-hospital mortality and clinical and microbiological outcomes among subgroups with different severity stratification criteria. Kaplan–Meier statistics were used to estimate 90-day mortality of the entire study population first stratified by subgroups followed by colistin-based therapy assignment. Statistical analysis was considered to be significant when the p value was < 0.05. All statistical analyses and visualization were performed using MedCalc Statistical Software version 19.0.7 (MedCalc Software bvba, Ostend, Belgium; https://www.medcalc.org; 2019) and Python packages Panda, NumPy, Matplotlib, and Scikit-learn (Python Software Foundation).

### Ethics approval and consent to participate

The study was approved by the ethics committee of each participating institution (Institutional Review Board [IRB] of Taipei Veterans General Hospital, IRB number: 2018-03-001CC; IRB of Tri-Service General Hospital, IRB number: 1-107-05-054; IRB of China Medical University Hospital, IRB number: CMUH107-REC3-052; IRB of Taichung Veterans General Hospital, IRB number: IRB CE18100A; IRB of Kaohsiung Medical University Hospital, IRB number: KMUHIRB-E(I)-20180141). Informed consent was waived because of the retrospective nature of the study. The study was performed in accordance with the Declaration of Helsinki concerning the ethical principles for medical research.

## Results

### Demographic characteristics

Of the 690 critically ill patients with nosocomial pneumonia caused by CRGNB, 458 were male and 232 were female, with a median age of 73.7 years. The most common comorbidity was hypertension (54.3%), followed by diabetes mellitus (35.9%), chronic lung disease (18.4%), chronic kidney disease (16.4%), and cerebral vascular disease (16.2%). With respect to pathogen identified in the study, 543 (78.7%) were *A. baumannii*, 90 (13%) were *P. aeruginosa*, 41 (5.9%) were *Klebsiella pneumoniae*, and 16 (2.3%) were *Enterobacter species*. The median length of stay (LOS) in the hospital prior to pneumonia onset was 13 days (IQR, 6–26 days). The median APACHE II and SOFA score on the day of pneumonia diagnosis were 22.0 (IQR, 17.0–27.0) and 7.0 (IQR, 5.0–10.0), respectively. Regarding the type of supportive care, most patients received mechanical ventilation (n = 650, 94.2%) and about half of the patients required vasopressor therapy (n = 320, 46.4%). A total of 293 (42.5%) patients received definitive therapy with colistin, of which only 46 (15.7%) patients received a combination of intravenous and inhaled forms.

### Prognostic factors for in-hospital mortality

Table 1 summarizes the comparison of demographic characteristics for patient mortality and survival. The overall in-hospital mortality was 46.1%. When compared with surviving patients, the deceased had a higher incidence of liver disease (11.3% vs. 7.0%, p = 0.048), chronic kidney disease (19.5% vs. 13.7%, p = 0.041), vasopressor therapy (65.7% vs. 29.8%, p < 0.001), continuous renal replacement therapy (24.8% vs. 9.7%, p < 0.001), longer LOS prior to pneumonia onset (17.0 days vs. 10.0 days, p < 0.001), and higher SOFA score (9.0 vs. 6.0, p < 0.001). Furthermore, smoking habits (13.8% vs. 20.7%, p = 0.018), cerebral vascular disease (13.2% vs. 18.8%, p = 0.047), and escalation of therapy (43.1% vs. 51.9%, p = 0.021) were significantly less frequent among the patients with mortality than the surviving patients. Overall, the multivariate analysis showed that in-hospital mortality was significantly associated with a LOS longer than 9 days prior to pneumonia onset (OR 2.18, 95% confidence interval (CI) 1.53–3.10, p < 0.001), a SOFA score on day of pneumonia more than 7 (OR 2.36, 95% CI 1.65–3.37, p < 0.001), supportive care with vasopressor therapy (OR 3.21, 95% CI 2.26–4.56, p < 0.001), and escalation of antimicrobial therapy (OR 0.71, 95% CI 0.50–0.99, p = 0.045), as depicted in Table 2.

**Table 1:** Demographic characteristics of the 690 analyzed patients according to in-hospital mortality.

	Total (n = 690)	Mortality (n = 318)	Survival (n = 372)	p value
Age, median (IQR), y	73.7 (61.4–83.5)	73.7 (62.1–85.1)	73.6 (60.8–82.5)	0.142
Male, No. (%)	458 (66.4)	212 (66.7)	246 (66.1)	0.882
Body mass index, median (IQR), kg/m^2^	23.0 (20.1–26.2)	22.5 (20.0–25.8)	23.4 (20.3–26.3)	0.061
**Personal history, No. (%)**				
Smoking habit	121 (17.5)	44 (13.8)	77 (20.7)	0.018
Alcohol consumption	131 (19.0)	58 (18.2)	73 (19.6)	0.644
**Comorbidities, No. (%)**				
Malignancy	93 (13.5)	50 (15.7)	43 (11.6)	0.111
Liver disease	62 (9.0)	36 (11.3)	26 (7.0)	0.048
Heart failure	82 (11.9)	42 (13.2)	40 (10.8)	0.321
Hypertension	375 (54.3)	167 (52.5)	208 (55.9)	0.372
Cerebral vascular disease	112 (16.2)	42 (13.2)	70 (18.8)	0.047
Chronic kidney disease	113 (16.4)	62 (19.5)	51 (13.7)	0.041
Chronic lung disease	127 (18.4)	63 (19.8)	64 (17.2)	0.379
Diabetes mellitus	248 (35.9)	107 (33.6)	141 (37.9)	0.246
Ventilator-associated, No. (%)	484 (70.1)	220 (69.2)	264 (71.0)	0.610
**Type of ICU, No. (%)**				0.164
Medical	441 (63.9)	215 (67.6)	226 (60.8)	
Cardiovascular	34 (4.9)	15 (4.7)	19 (5.1)	
Surgical	215 (31.2)	88 (27.7)	127 (34.1)	
LOS prior to pneumonia onset, median (IQR), days	13.0 (6.0–26.0)	17.0 (8.0–32.0)	10.0 (5.0–20.0)	< 0.001
**Microbiological organisms, No. (%)**				0.579
*Acinetobacter baumannii*	543 (78.7)	249 (78.3)	294 (79.0)	
*Pseudomonas aeruginosa*	90 (13.0)	43 (13.5)	47 (12.6)	
*Klebsiella pneumoniae*	41 (5.9)	21 (6.6)	20 (5.4)	
*Enterobacter species*	16 (2.3)	5 (1.6)	11 (3.0)	
**Severity score, median (IQR)**				
APACHE II score	22.0 (17.0–27.0)	23.0 (17.0–28.0)	22.0 (17.0–27.0)	0.194
SOFA score	7.0 (5.0–10.0)	9.0 (7.0–11.0)	6.0 (4.0–9.0)	< 0.001
**Supportive care, No. (%)**				
Vasopressor therapy	320 (46.4)	209 (65.7)	111 (29.8)	< 0.001
Mechanical ventilation	650 (94.2)	296 (93.1)	354 (95.2)	0.244
Hemodialysis	103 (14.9)	49 (15.4)	54 (14.5)	0.743
Continuous renal replacement therapy	115 (16.7)	79 (24.8)	36 (9.7)	< 0.001
Extracorporeal membrane oxygenation	16 (2.3)	11 (3.5)	5 (1.3)	0.066
**Antimicrobial therapy, No. (%)**				
Escalation of therapy, No. (%)	330 (47.8)	137 (43.1)	193 (51.9)	0.021
Definitive therapy with colistin, No. (%)	293 (42.5)	134 (42.1)	159 (42.7)	0.873
Intravenous alone	96 (13.9)	51 (16.0)	45 (12.1)	0.136
Inhaled alone	151 (21.9)	61 (19.2)	90 (24.2)	0.113
Combination	46 (6.7)	22 (6.9)	24 (6.5)	0.807

*APACHE II* Acute Physiology and Chronic Health Evaluation II, *ICU* intensive care unit, *IQR* interquartile range, *LOS* length of stay, *SOFA* Sequential Organ Failure Assessment.

**Table 2:** Multivariate analysis of prognostic factors for in-hospital mortality.

Variables	Univariate analysis		Multivariate analysis	
	Odds ratio (95% CI)	p value	Odds ratio (95% CI)	p value
Smoking habit	0.62 (0.41–0.92)	0.019		
**Comorbidities**				
Liver disease	1.70 (1.00–2.88)	0.049		
Cerebral vascular disease	0.66 (0.43–1.00)	0.047		
Chronic kidney disease	1.52 (1.02–2.29)	0.042		
LOS prior to pneumonia onset > 9 days	2.39 (1.74–3.28)	< 0.001	2.18 (1.53–3.10)	< 0.001
SOFA score on day of pneumonia > 7	3.52 (2.57–4.82)	< 0.001	2.36 (1.65–3.37)	< 0.001
**Supportive care**				
Vasopressor therapy	4.51 (3.27–6.21)	< 0.001	3.21 (2.26–4.56)	< 0.001
Continuous renal replacement therapy	3.09 (2.01–4.73)	< 0.001		
Escalation of therapy	0.70 (0.52–0.95)	0.021	0.71 (0.50–0.99)	0.045

*CI* confidence interval, *LOS* length of stay, *SOFA* Sequential Organ Failure Assessment.

### The pattern of organ involvement

**Figure 1:**
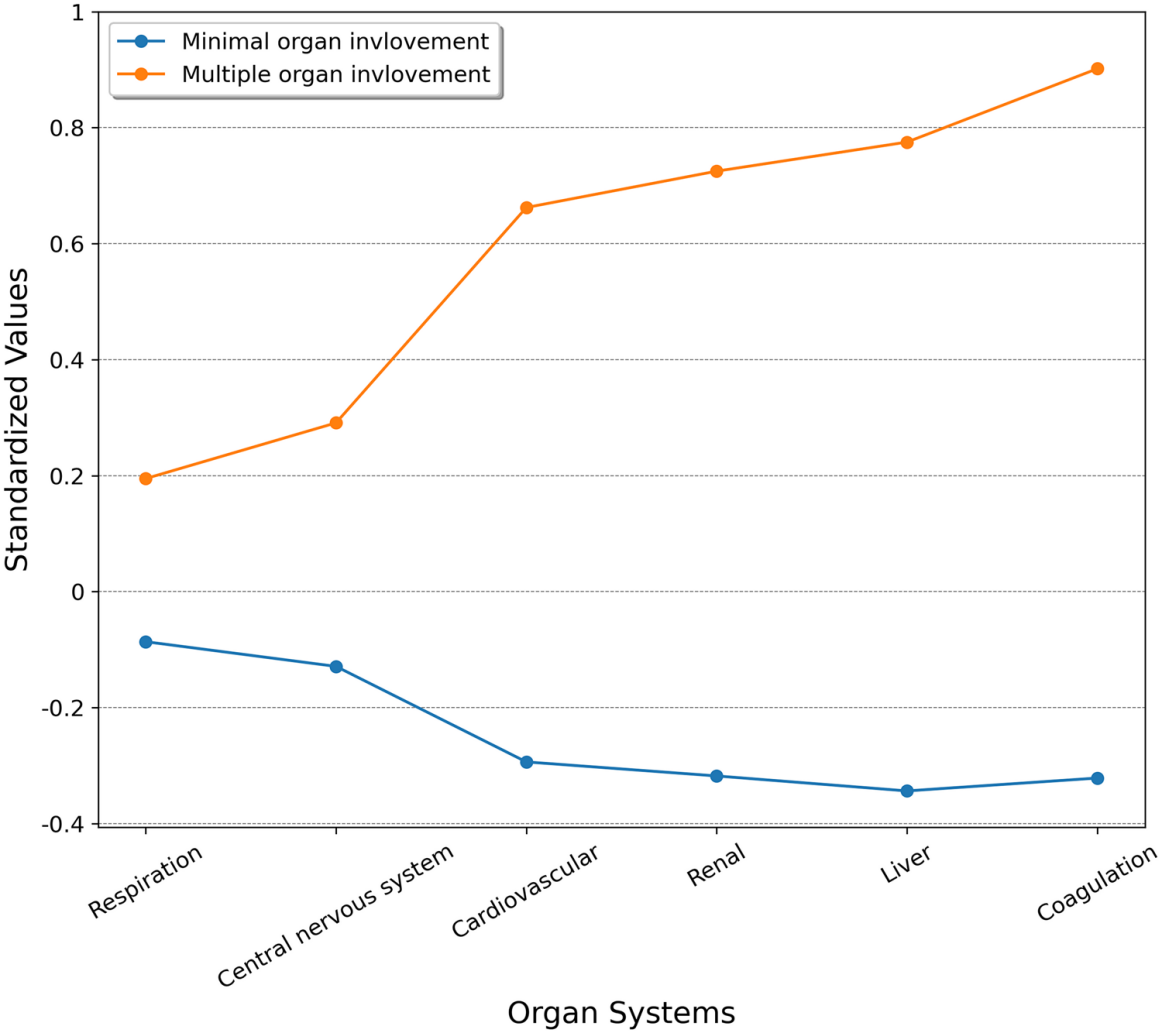
Differences in standardized values of each SOFA subscore by subtype. The y-axis represents standardized values, in which all means were scaled to 0 and standard deviations to 1. *SOFA* Sequential Organ Failure Assessment.

All patients had complete SOFA subscore data and were included in the analysis. The evaluation of k-means clustering performance suggested that a two-cluster model was the best option (Additional file 1: eFigure 1). As shown in Fig. 1, patients in cluster 1 had more organ dysfunction (more coagulation and liver, cardiovascular, and renal dysfunction) compared with patients in cluster 2. Therefore, we denoted the clusters as the multiple organ involvement subtype (cluster 1) and the minimal organ involvement subtype (cluster 2). Cluster 1 had higher in-hospital mortality, lower clinical cure, and lower microbiological eradication rate at day 14 than cluster 2. The detailed SOFA subscore data and outcome evaluation of the two clusters are presented in Table 3.

**Table 3:** SOFA subscore data and clinical outcomes of the two clusters.

	Cluster 1: Multiple organ involvement (n = 212)	Cluster 2: Minimal organ involvement (n = 478)	p value
SOFA score, mean (SD)	12.0425 (2.7577)	5.9435 (2.2070)	< 0.001
Respiration, mean (SD)	1.7547 (1.3085)	1.3891 (1.2834)	< 0.001
Coagulation, mean (SD)	1.9434 (1.1547)	0.4665 (0.7756)	< 0.001
Liver, mean (SD)	1.2736 (1.3942)	0.1464 (0.4664)	< 0.001
Central nervous system, mean (SD)	3.1321 (0.8662)	2.7176 (1.0124)	< 0.001
Cardiovascular, mean (SD)	1.6179 (1.5517)	0.4414 (0.8366)	< 0.001
Renal, mean (SD)	2.3208 (1.5117)	0.7824 (1.1775)	< 0.001
**Outcomes**			
In-hospital mortality, No. (%)	137 (64.6)	181 (37.9)	< 0.001
Cure/improvement at day 14, No. (%)	99 (46.7)	285 (59.6)	0.002
Eradication at day 14, No. (%)	39 (18.4)	125 (26.2)	0.027

*SD* standard deviation, *SOFA* Sequential Organ Failure Assessment.

### Antimicrobial therapy

Most patients received combination therapy (n = 500, 72.5%) throughout the study. A total of 330 (47.8%) patients received escalation therapy. Of the 465 antimicrobial prescriptions for escalation therapy, the most common agents were inhaled colistin (n = 126, 27.1%), followed by tigecycline (n = 82, 17.6%), intravenous colistin (n = 79, 17.0%), and cefoperazone-sulbactam (n = 48, 10.3%). Furthermore, of the 1084 antimicrobial prescriptions for definitive therapy, the most common agents were inhaled colistin (n = 197, 18.2%), followed by tigecycline (n = 144, 13.3%), intravenous colistin (n = 142, 13.1%), cefoperazone-sulbactam (n = 90, 8.3%), and meropenem (n = 90, 8.3%). Overall, a total of 293 (42.5%) patients received colistin-based therapy (including definitive or escalated therapy with either intravenous or inhaled colistin regimen), of which 181 (61.7%) patients were treated for the purpose of escalation of therapy.

**Figure 2:**
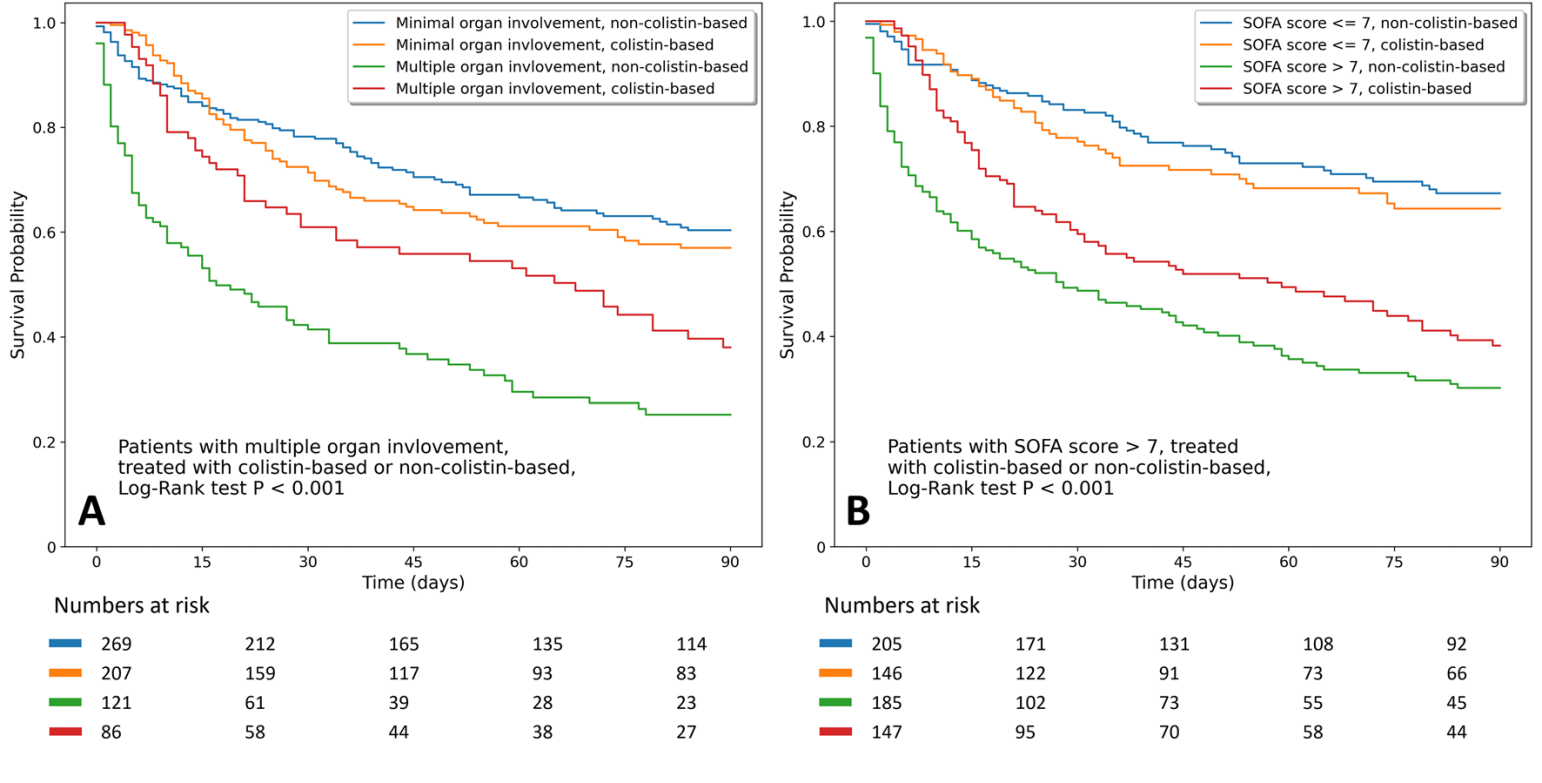
(**a**) Kaplan–Meier survival curves for 90-day mortality stratified by pattern of organ involvement (minimal and multiple) and antimicrobial therapy (colistin-based and non-colistin-based). (**b**) Kaplan–Meier survival curves for 90-day mortality stratified by SOFA score (> 7 and ≤ 7) and antimicrobial therapy (colistin-based and non-colistin-based). *SOFA* Sequential Organ Failure Assessment.

**Figure 3:**
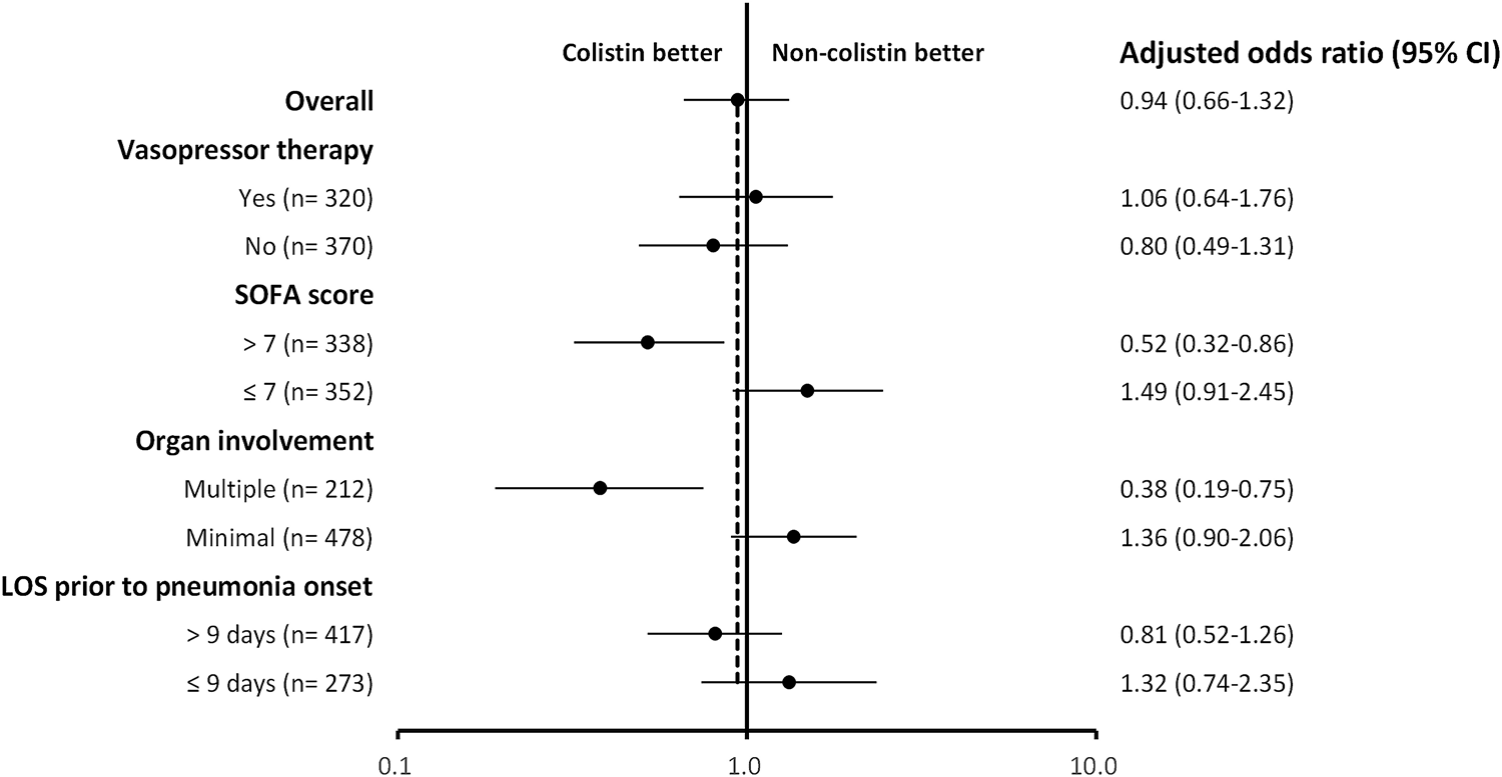
Forest plot of adjusted odds ratios for in-hospital mortality in the colistin-based group versus non-colistin-based group. Multivariate analysis adjusted for age, gender, smoking habit, comorbidities with liver disease, cerebral vascular disease, chronic kidney disease, LOS prior to pneumonia onset, SOFA score, supportive care with vasopressor therapy, and continuous renal replacement therapy. *CI* confidence interval, *LOS* length of stay, *SOFA* Sequential Organ Failure Assessment.

For critically ill patients with nosocomial pneumonia caused by CRGNB, colistin-based therapy did not provide a better survival benefit than non-colistin-based therapy (54.3% vs. 53.7%, p = 0.873). Colistin-based therapy showed different influence on survival outcome in the subgroup analysis, as shown in the Kaplan–Meier curve of 90-day mortality across patients with colistin-based therapy with the pattern of organ involvement (Fig. 2a) and SOFA score (Fig. 2b). Patients with multiple organ involvement and a SOFA score of more than 7 had better survival outcomes with colistin-based therapy than non-colistin-based therapy (adjusted OR 0.38, 95% CI 0.19–0.75, p = 0.006; adjusted OR 0.52, 95% CI 0.32–0.86, p = 0.011, respectively). The influence of colistin-based therapy in patients with different severity stratification criteria is depicted in Fig. 3. Similar findings were also observed regarding the clinical and microbiological outcomes (Additional file 1: eFigure 2).

## Discussion

Carbapenem-Resistant Gram-Negative Bacteria are a prevalent hospital-acquired infection with poor prognosis and high mortality, especial in the ICU^2,3^. More data on CRGNB infections in the ICU are required to explore prognostic factors and outcomes to guide therapeutic decisions. In this multicenter ICU study of nosocomial CRGNB pneumonia, significant predictors of in-hospital mortality included LOS in hospital prior to pneumonia of longer than 9 days, a SOFA score of more than 7, supportive care with vasopressor therapy, and escalation of antimicrobial therapy. Subgroup analysis showed that patients with multiple organ involvement or a SOFA score greater than 7 had a better survival outcome when receiving colistin-based therapy.

Through nationwide surveillance in Taiwan since 2011, an increased resistance to carbapenem has been demonstrated in *A. baumannii*, *K. pneumoniae,* and *P. aeruginosa* from 69.8 to 75.3%, 10.5 to 44.4% and 17.7 to 22.6%, respectively^23^. Previous studies have demonstrated that risk factors, such as carbapenem consumption, LOS, diabetes mellitus, multiple lobar involvement, and prior exposure to antimicrobial therapy, are associated with the acquisition of CRGNB infection^24–27^. Moreover, the mortality rate of nosocomial pneumonia caused by CRGNB is also high. By pooling data from previous clinical studies, the mortality rate was in excess of 40%, even in the subgroup of patients receiving colistin-based combination therapy^9,10^. As the only prognostic factor amenable to intervention, appropriateness of empirical antimicrobial therapy has been extensively evaluated. Despite the promising results from early observational studies^7,8,28^, a more recent randomized control trial failed to demonstrate any benefit from empiric treatment with colistin in an attempt to improve its appropriateness for ventilator-associated pneumonia^29^. There are concerns regarding the potential for developing nephrotoxicity and the emergence of antimicrobial resistance following colistin treatment^30,31^. Considering the poor prognosis and controversial role of colistin-based therapy for nosocomial pneumonia caused by CRGNB, our study aimed to investigate prognostic factors to improve therapeutic decisions.

In this study, we present a multicenter retrospective study of 690 critically ill patients with nosocomial pneumonia caused by CRGNB. Consistent with previous studies^9,10,22^, about 80% of patients had late-onset nosocomial pneumonia (defined as pneumonia after five or more days of hospitalization) with the median LOS prior to pneumonia onset of 13 days. Approximately 46% of patients eventually died in hospital. Moreover, we noted that LOS in hospital following pneumonia diagnosis was prolonged, with a median of 28 days. These results reinforce the importance that while treating patients with nosocomial pneumonia caused by CRGNB, clinicians should be aware of the high mortality and economic burden. Every effort should be made to prevent nosocomial cross-transmission between patients.

Using multivariate logistic regression analysis, we identified that a LOS in hospital longer than 9 days prior to pneumonia onset, a SOFA score of more than 7, and supportive care with vasopressor therapy negatively affected the prognosis. Escalation of antimicrobial therapy positively influenced patient survival. Timely escalation of therapy is an important factor since it is currently the only intervening factor shown to improve clinical outcome.

Previous studies demonstrated that prolonged LOS prior to pneumonia onset, with a cut-off point ranging from 4 to 7 days, was associated with the risk of acquisition of potentially resistant pathogens, which resulted in increased mortality^1,32,33^. In our study, which focused on patients with nosocomial pneumonia caused by CRGNB, we found that a prolonged hospital stay of more than 9 days was an independent prognostic factor, regardless of comorbidity and disease severity, with a sensitivity, specificity, and area under the receiver-operation characteristics curve (AUROC) of 71.4%, 48.9%, and 0.63, respectively. This phenomenon might be explained by the fact that patients with a prolonged hospital stay tend to be frailer and have greater disease complexity, resulting in elevated mortality^34^.

Septic shock and multiple organ failure are considered to be common and deadly complications of pneumonia^35^, especially in cases caused by CRGNB^8^. Consistent with previous studies, we noted that a high SOFA score and vasopressor requirement were significant risk factors for mortality. The present study demonstrated that a SOFA score of more than 7 was associated with increased mortality with a sensitivity, specificity, and AUROC of 65.4%, 65.1%, and 0.70, respectively. To identify the pattern of organ involvement, we applied a k-means algorithm using SOFA subscore data. K-means is one of the most widely used clustering techniques in unsupervised learning^36^. Regarding the complex and heterogeneous nature of critically ill patients, phenotype identification has been well introduced in previous studies, especially in the field of sepsis^37,38^ and acute respiratory distress syndrome^39^. In our study, by using a simple algorithm and six parameters, without other clinical and biological characteristics, we identified two distinct subtypes with different disease trajectories and outcomes. This finding suggests that evaluating individual SOFA subscores rather than the total score alone could be helpful in clinical practice for severity stratification.

In the present study, escalation of therapy was recognized as the only modifiable factor to decrease in-hospital mortality. Due to the high prescription rate for escalation therapy, we further evaluated the influence of colistin-based therapy on patient outcomes. Although colistin is often considered to be a reliable option for the treatment of CRGNB infection, previous meta-analyses only showed significant effects for clinical cure and microbiological eradication but not for mortality^10,20^, which was similar to our findings. As different severity stratification criteria may be a factor that influences therapeutic response and clinical outcome, we further analyzed and demonstrated that colistin-based therapy was associated with better survival outcomes in patient subgroups with a SOFA score of more than 7 and multiple organ involvement. These results reinforce that while treating critically ill patients with nosocomial pneumonia in the setting of high prevalence of CRGNB, high SOFA scores and the presence of multiple organ involvement should not be ignored by clinicians. In this condition, timely escalation of colistin-based therapy may improve patient outcomes.

Our multicenter design overcomes potential single-center bias and strengthens the external validity of our findings. In addition, we studied a large critically ill population from five tertiary teaching hospitals covering different regions of Taiwan, supporting its high representative reliability. However, our study has several limitations. First, given the nature of retrospective analysis, missing data were unavoidable, and the prescription pattern of antimicrobial therapy was heterogeneous and complex. Therefore, it is impossible to compare various treatment strategies directly and to determine the most effective therapy for nosocomial pneumonia caused by CRGNB. Second, in vitro susceptibility for colistin was not tested at all the participating hospitals. In addition, reporting the upper limit values of MIC for meropenem/doripenem was not all consistent. Hence, it is impractical to assign patients based on inappropriateness of therapy and evaluate its influence on patient outcomes. Third, as an important complication of pneumonia, acute respiratory distress syndrome was not recorded and could be a confounding factor in this study. Fourth, due to lack of ground-truth reference, k-means clustering performance was only evaluated by internal validation. Further prospective studies should be performed to assess external validity and confirmation of robustness.

## Conclusions

Despite advances in critical care, mortality due to nosocomial pneumonia caused by CRGNB remains high. Prolonged LOS prior to pneumonia onset, high SOFA score, vasopressor requirement, and timely escalation of antimicrobial therapy were independent prognostic factors for in-hospital mortality. Despite the lack of benefit in the entire study population, colistin-based therapy was associated with better survival outcomes in patient subgroups with a SOFA score of more than 7 and multiple organ involvement.

## Supplementary Information

Supplementary Information.

## Data Availability

The datasets used and/or analysed during the current study are available from the corresponding author on reasonable request.

## Abbreviations

ALAT: Asociacion latinoamericana del torax
AUROC: Area under the receiver-operation characteristics curve
CI: Confidence interval
CRGNB: Carbapenem-resistant gram-negative bacteria
ERS: European Respiratory Society
ESCMID: European Society of Clinical Microbiology and Infectious Diseases
ESICM: European Society of Intensive Care Medicine
ICU: Intensive care unit
IRB: Institutional Review Board
MIC: Minimum inhibitory concentration
SD: Standard deviation
VAP: Ventilator-associated pneumonia
