# Supplementary material for: Clinical outcome of nosocomial pneumonia caused by Carbapenem-resistant gram-negative bacteria in critically ill patients: a multicenter retrospective observational study
Source: Sci Rep. 2022 May 7;12:7501. doi: 10.1038/s41598-022-11061-7 (PMC9079069; doi:10.1038/s41598-022-11061-7)
Supplement: Supplementary file 1 — Supplementary Information. [file 41598_2022_11061_MOESM1_ESM.pdf]

<u>Supplement</u>

Clinical Outcome of Nosocomial Pneumonia Caused by Carbapenem-Resistant Gram-Negative Bacteria in Critically Ill Patients: A Multicenter Retrospective Observational Study

Chen et al.

eFigure 1

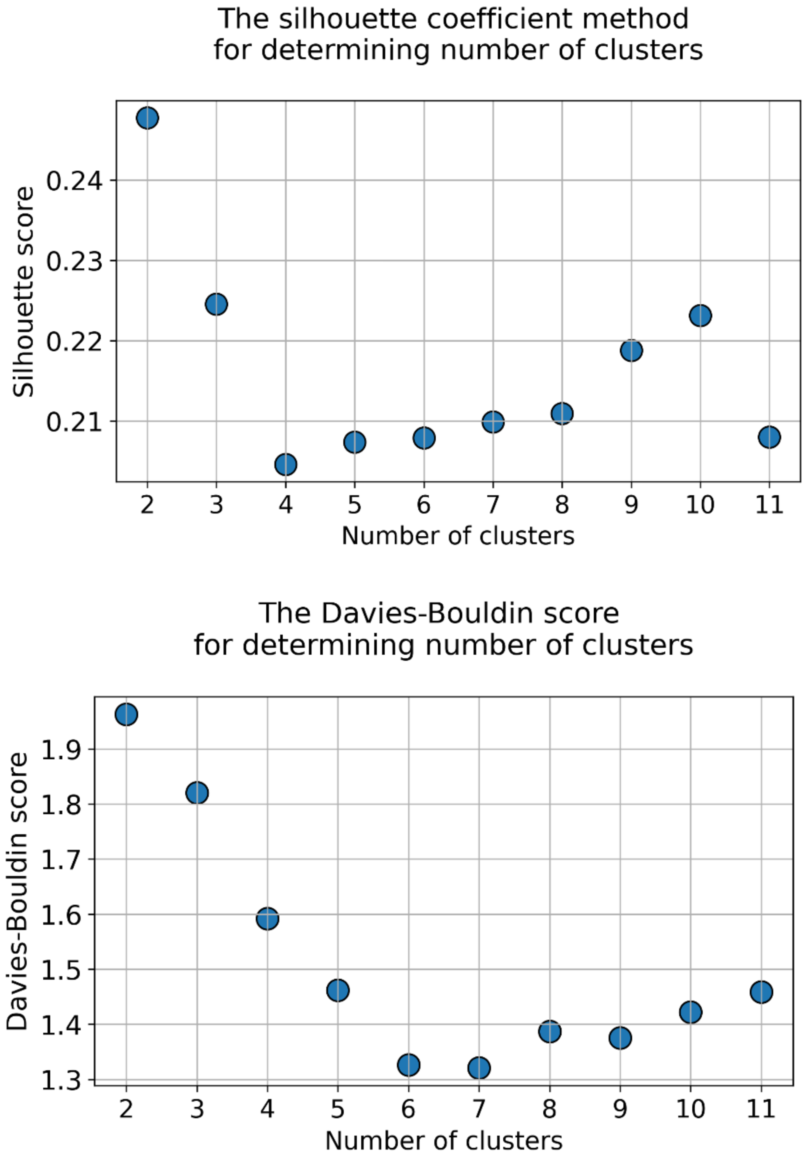

eFigure 1: Internal validation measures for determining number of clusters.

eFigure 2

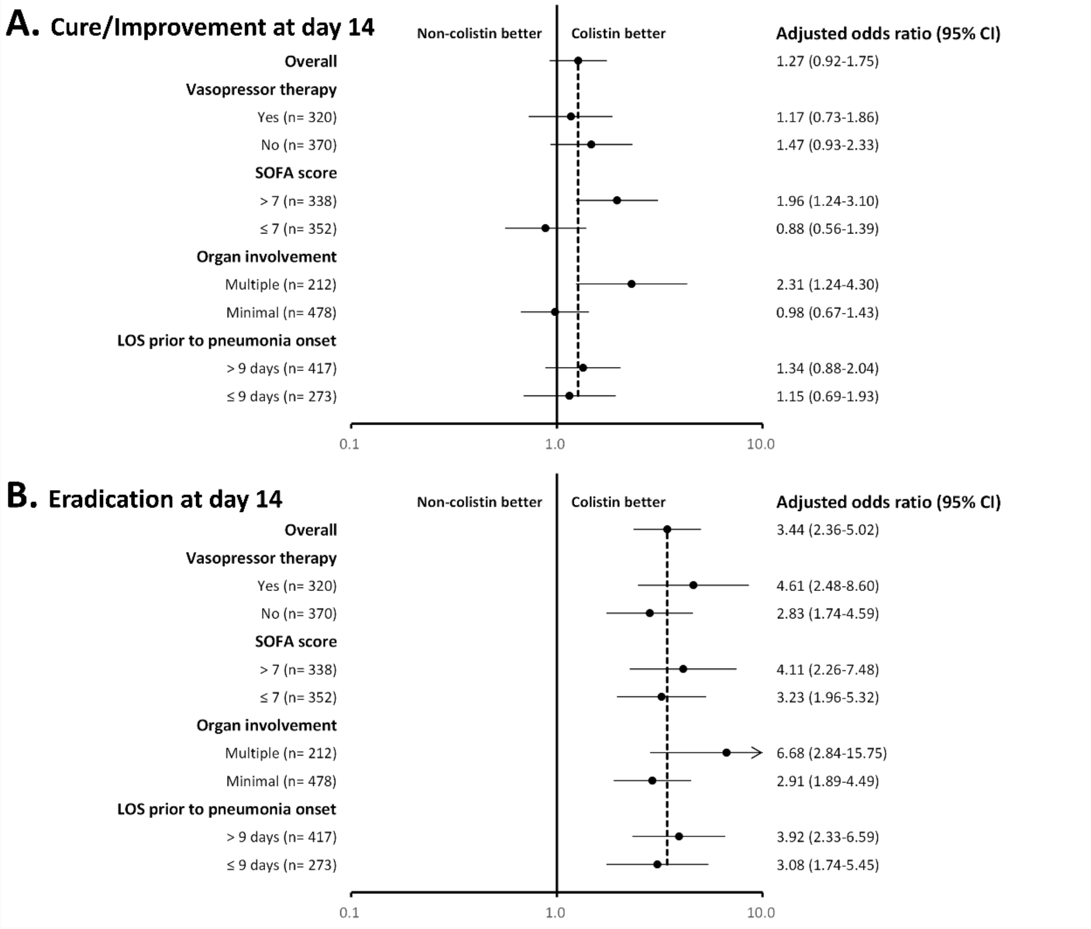

eFigure 2: (a) Forest plot of adjusted odds ratios for clinical response in the colistin-based group versus non-colistin-based group. (b) Forest plot of adjusted odds ratios for microbiological eradication in the colistin-based group versus non-colistin-based group.

Multivariate analysis adjusted for age, gender, smoking habit, comorbidities with liver disease, cerebral vascular disease, chronic kidney disease, LOS prior to pneumonia onset, SOFA score, supportive care with vasopressor therapy, and continuous renal replacement therapy.

Abbreviations: CI, confidence interval; LOS, length of stay; SOFA, Sequential Organ Failure Assessment.
